# Family psychoeducation to support patients with psychotic illness: two-year outcomes from a pre–post longitudinal pilot study

**DOI:** 10.3389/fpsyt.2026.1724396

**Published:** 2026-05-14

**Authors:** Roxoliana Tsisar, Kristen E. Zentner, Katherine Shettell, Adam Abba-Aji, Melanie Robles

**Affiliations:** 1 Department of Psychiatry, University of Alberta, Edmonton, AB, Canada; 2 Department of Psychology, University of British Columbia - Okanagan, Kelowna, BC, Canada; 3 Clinical Supports and Services, Addiction and Mental Health, Recovery Alberta, Edmonton, AB, Canada; 4 Department of Family Medicine, University of Alberta, Edmonton, AB, Canada

**Keywords:** early intervention, family intervention, family psychoeducation, psychosis, schizophrenia

## Abstract

**Background:**

Psychoeducation for families of young adults with psychosis is an evidence-based intervention that alleviates carer burden. The implementation of programming is limited, leaving family carers shouldering a heavy burden without appropriate support.

**Objective:**

This pre-post longitudinal pilot study evaluated the preliminary outcomes of a psychoeducational group intervention for family carers of young adults with psychosis, aimed at building skills and reducing carer burden to support recovery in their loved ones.

**Methods:**

The intervention, co-developed and co-facilitated by healthcare professionals and individuals with family lived experience, was delivered in Edmonton, Canada. Participants (*n*= 13) completed the Family Burden Interview Schedule (FBIS) at pre-intervention, post-intervention, and at 6, 12, and 24-month follow-up. Linear mixed models assessed burden scores over time.

**Results:**

The overall model of total burden did not reach statistical significance. Exploratory *post-hoc* comparisons indicated a significant total burden reduction from pre-intervention to 6-months (*p* = 0.032), with no other significant changes. The overall family interaction burden subscale model showed no significant effect of time. Exploratory *post-hoc* analyses indicated a decrease in family interaction burden from pre- to post-intervention (*p* = 0.026) and to 6- months (*p* = 0.032), with no other significant changes.

**Conclusion:**

This pilot study provides preliminary and hypothesis-generating findings suggesting a co-produced, skills- and knowledge-based psychoeducational intervention may be associated with reductions in carer burden, particularly in the domain of family relations. Given the small sample size, further research with sufficient statistical power is warranted to evaluate the long-term impact and accessibility of the intervention and inform its integration into early psychosis care.

## 1 Introduction

Family carers of young adults affected by psychosis provide extensive practical and emotional support, and advocate for their loved ones through periods of crisis and recovery that may persist across the lifespan (1). The profound burden associated with this caregiving role has a significant effect on the family carer’s wellness and functioning. In a sample of carers supporting a loved one with severe mental illness, 39.7% met criteria for a complex grief reaction, which was associated with higher rates of negative health outcomes, including post-traumatic stress, depression, and poorer physical health (2). As well, carers experience social isolation and a reduced quality of life (3). Validating and supporting family carers in their roles mitigates this heavy carer burden and risk of burnout and strengthens carers’ positive influence on their loved one’s recovery (4).

The importance of developing family-integrated approaches to care for individuals affected by psychotic illness emerged in parallel with the replacement of institution-based care by community-based systems, which in practical terms are highly predicated on the extensive support provided by family (4). Expressed emotion research, which demonstrated that family environments have a strong influence on relapse risk, brought early interest to collaborative family involvement (5, 6). Open Dialogue and other dialogic and recovery oriented models emphasize the values of transparency, shared decision making and collaborative relationships in involving families and wider support networks in early and ongoing psychosis care (7, 8). Family interventions that incorporate communication strategies focused on alliance building and acceptance-based approaches which highlight cognitive flexibility and tolerance of uncertainty, have increasing empirical support (9–11). Together, these foundations support family-centered approaches that are relational, non-blaming, and responsive to the lived realities of psychosis.

Worldwide clinical practice guidelines for early psychosis care recommend family interventions as a strategy to fortify family carers’ position within the triad of care, a recovery model promoting collaboration between a young adult affected by psychosis, their supporting family and their clinical care team (12–15). Despite robust evidence that supports these recommendations, implementation remains challenged and carers continue to experience isolation and lack of access to credible resources, including skills and education necessary to effectively support their loved ones (16, 17). This constitutes a gap in family-centered care and calls for the development and implementation of psychoeducational programming that is evidence-based, informed by the lived experience of carers, and easily accessible within the health system.

It is important that psychoeducational programs address unique challenges that carers face with respect to how symptoms of psychosis affect their relationship with their young adult. Anosognosia, the neurological inability to recognize symptoms of one’s illness, creates a distinctive barrier to treatment in affected young adults and substantial stress for family carers (18). Anosognosia is a symptom of psychotic illness and is present in at least 60% of individuals with schizophrenia (19, 20). The presence of anosognosia is among the factors most predictive of poorer long-term recovery from psychosis, and contributes to the disengagement of young adults from treatment and from supports (19, 20). Psychoeducational programming hones carer skills and knowledge that can help preserve and enhance relationships even in the presence of difficult symptoms including anosognosia, aiding the carers to supportively navigate psychotic illness in partnership with their loved one (21).

High-level psychoeducational programs also harness the synergistic expertise of clinical professionals and individuals with family lived experience (22). Interventions developed and delivered collaboratively meet carers compassionately and respectfully in their immediate need and offer hope, relevant information and practical tools to support their loved ones’ recovery, manage personal stress, and reinforce their essential role within the triad of care (13, 23, 24). The sharing of stories of family carers further along in the caregiving journey is a powerful tool that increases insight and resilience in the carers who are at an earlier stage (16). Co-production throughout the lifecycle of an intervention has been linked to improved relevance, acceptability, and overall effectiveness (22).

The expected effect of implementing evidence-based programming for carers is a reduction in symptom relapse, enhanced treatment adherence, and improved social and occupational functioning in the young adults affected by psychosis, alongside psychosocial benefits for the carers themselves (21, 23, 25). While some programs have been developed to fill this gap, rigorous evaluations of the sustained impact of these interventions over extended periods of time are limited. This study aimed to evaluate preliminary evidence of the effectiveness of a co-produced, evidence-informed psychoeducational group intervention in reducing family burden and improving family relationship dynamics over a 24-month period. The current paper presents the evaluation of data gained from the pilot study. The pilot study served as a precursor to a broader Phase II evaluation of the intervention, which is currently underway in a larger cohort of participants recruited from the Edmonton Zone (23).

## 2 Methods

This pilot study employed a longitudinal pre–post design, without a control group, with follow-up assessments immediately post-intervention and at 6 months, 12 months, and 24 months post-intervention to evaluate the impact of a co-produced psychoeducational intervention on caregiver burden (26). The intervention was delivered from May to June 2022.

### 2.1 Participants

Fourteen family carers (*M* age = 53.0, SD = 8.6) of young adults (*M* age = 24.0, SD = 2.7) were enrolled in the pilot study. One participant was lost to attrition and excluded from analyses, resulting in a retention of 92.9% of participants at 2 years. Inclusion criteria required that 1) the carer was a family supporter of a young adult aged 17–27 who had been admitted to or discharged from a psychiatric inpatient unit within the Edmonton Zone, in the province of Alberta, Canada, within the preceding 12 months, and 2) the carer had proficiency in English. Participants were recruited through community-based support groups, inpatient care teams, hospital-based family information sessions, and hospital bulletin board postings. A summary of participant demographics is presented in Appendix 1.

### 2.2 Intervention

The psychoeducational intervention was delivered to the 14 enrolled family carers in a closed, group-based format via secure video conferencing in 2-hour sessions over nine consecutive weeks. The curriculum included nine expert-reviewed, evidence-informed, skills and knowledge-focused modules as described in the study protocol (26). Each module was delivered to the group by facilitator dyads with synergistic professional and family lived experience expertise. Four of the modules focused on skills training, specifically the LEAP^©^ communication technique (Listen, Empathize, Agree, Partner) and brief interventions from ACTp for carers (Acceptance and Commitment Therapy) (9, 11, 27, 28). Three modules taught respectively about the family’s emotional journey, a biomedical model of psychosis and concordant illness (psychosis and addictions). The introductory module was designed to create a safe and confidential space for learning within the closed group. The final module reviewed and integrated material and included a story of hope from an individual with family lived experience spanning long-term recovery. All sessions incorporated structured psychoeducational content along with interactive components, including skills practice, group discussion, and question periods.

### 2.3 Participant feedback and qualitative analysis

Direct participant feedback was collected anonymously at the midway point (Module 5), and at the conclusion of the course (Module 9) using REDCap (Research Electronic Data Capture), a secure, web-based software platform for data collection (29, 30). Eight participants completed the feedback surveys, contributing to a total of 118 feedback segments following segmentation into discrete feedback segments. Participants’ feedback was collected during class time using an open text-field questionnaire. The questionnaire included two open-ended questions for each of the nine modules: “What did you like about this session?” and “What could be done to improve this session in the future?”. Responses ranged in length from 3 words (“good information overall”) to 156 words.

Qualitative feedback, which consisted of brief comments about each module, was analyzed using a structured coding process. The first author segmented the participant responses into discrete feedback segments and then categorized these responses as either positive (strengths and perceived benefits of the intervention), or critical (areas for improvement). The first and second author reviewed the data segments to identify recurring themes. A set of analytic codes was developed inductively from these themes and discussed between coders to ensure consistency. The first author conducted coding by applying the coding framework across all feedback segments. The second author reviewed categorization to ensure consistency. The frequencies of codes were calculated to find prominence of the identified themes and inform intervention refinement.

### 2.4 Measures

The Family Burden Interview Schedule (FBIS-24) was used to measure the total burden of the family related to supporting their loved one with psychosis, and includes the following domains: financial, routines and activities, leisure, relationships, physical health and mental health (31). Previous literature shows high inter-rater reliability between clinicians and family carers for all items (r ≥0.87) (32).

#### 2.4.1 Analyses

Analyses were conducted to examine preliminary trends over time. A linear mixed model was run in Jamovi (version 2.3.28) to model the trajectory of family burden over time with time as a fixed effect and subjects as a random effect, at five time points: pre-intervention, post-intervention, 6-month, 12-month, and 24-month follow-up. Given the small sample size and preliminary nature of the analyses in the current pilot project, an alpha-level of 0.05 was used. Aligning with the intervention’s emphasis on improving intra-family communication, an additional linear mixed model was conducted using the Family Interaction subscale of the FBIS as the dependent variable.

## 3 Results

### 3.1 Participant feedback

A total of 118 feedback segments were coded across all the modules. Participant feedback indicated high acceptability of the psychoeducational class, describing the modules as relevant, supportive, and effective in strengthening their understanding of psychosis and its impact on families. Positive feedback themes included 1) Increased knowledge and understanding about psychosis, 2) Normalization and validation of caregiving experiences, 3) Emotional support and improved coping, 4) Practical tools, resources, and strategies, 5) Increased hope and a perspective shift on challenges, and 6) Learning through group interaction and family lived-experience contributions. Among the coded positive feedback, knowledge gains (36.8%) and group learning (20.1%) were the most frequently occurring positive codes. Critical feedback themes, reflecting reported areas for improvement, focused on 1) Requests for more module specific knowledge or resources, 2) Delivery or format optimization, and 3) Support for managing emotionally intense course content. Delivery optimization (41.9%) and requests for more specific resources (38.7%) were the most frequently occurring critical codes.

### 3.2 Family burden outcomes

Shapiro-Wilk tests of normality of residuals were conducted for each analysis, and all indicated no significant deviation from normality (*p* = ns). Descriptive statistics are presented in **Table 1**.

**Table 1:** Demographic Characteristics of Pilot Sample.

Characteristic		*n*	% of sample
Sex of Participant	Female	10	77%
	Male	3	23%
Ethnicity of Participant	Black	3	23%
	Indigenous	1	8%
	South Asian	1	8%
	White	8	62%
Sex of Young Adult with Psychosis	Female	2	15%
	Male	11	85%
Relationship of Young Adult to Participant	Child	10	77%
	Grandchild	2	15%
	Niece/Nephew	0	0%
	Sibling	1	8%
Participant Education	High School	1	8%
	College, CEGEP or other non-university certificate	7	54%
	University Diploma/certificate	4	31%
	Doctoral Degree	1	8%
Employment Status	Casual	1	8%
	Full-time	10	77%
	Self-employed	1	8%
	Retired	1	8%
Marital Status	Married	9	69%
	Divorced	2	15%
	Widowed	1	8%
	Other	1	8%
Participant Difficulty Meeting Basic Needs	Yes	1	8%
	No	12	92%
Presence of Other Supports	Yes	11	85%
	No	2	15%
Young Adults’ Living Situation	Within Family’s Home	9	69%
	Own Home	3	23%
	Other	1	8%

**Figure 1:**
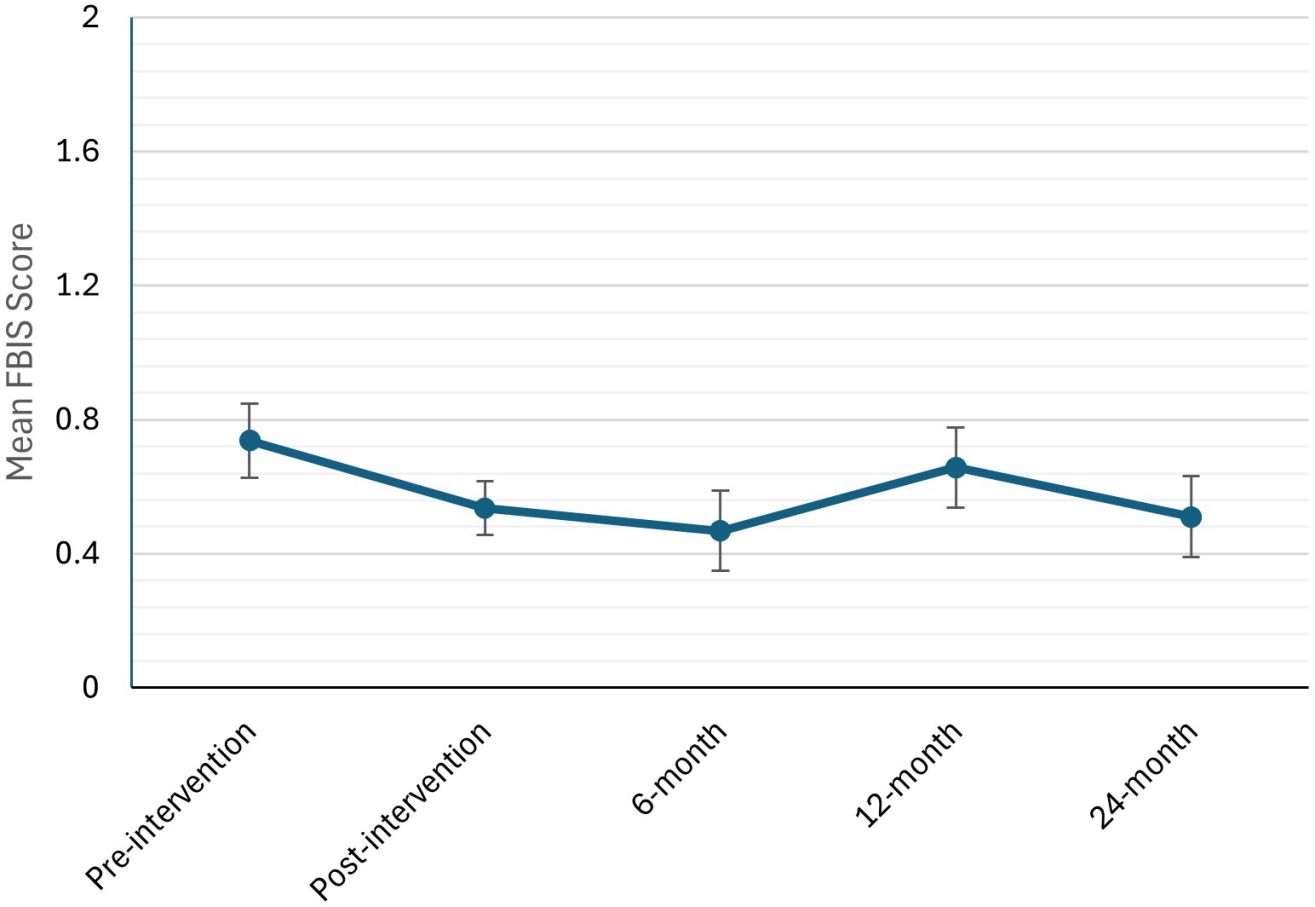
Mean total family burden interview schedule (FBIS) scores across five time points (pre-intervention, post-intervention, 6-, 12-, and 24-month follow-up). Error bars represent standard error of the mean.

The overall FBIS score model revealed a non-significant main effect of time, *F* (4, 48) = 1.69, *p* = 0.168, *R²* = 0.0609, indicating that total family burden did not change significantly across the study period (**Table 2**; **Figure 1**). Exploratory *post-hoc* comparisons with Bonferroni adjustment revealed a significant reduction in total burden from pre-intervention to 6-months post-intervention (*p* = 0.032). Changes from pre-intervention to post-intervention (*p* = 0.104), to 12-month follow-up (*p* = 0.516) and to 24-month follow-up (*p* = 0.068) were each not significant.

**Table 2:** Fixed effects of FBIS^a^ scores across 24-month follow up.

Variable	Estimate [CI]^b^	p^c^	R^2d^	AIC^e^	BIC^f^	LogLikel.^g^	ICC^h^
Model 1			0.00	58.419	68.239	-27.858	0.370
Intercept	0.581 [0.428, 0.735]	<0.001					
Model 2			0.0609	59.575	88.917	-29.848	0.386
Intercept	0.581 [0.428, 0.735]	<0.001					
Time	-0.105 [-0.274, 0.064]	0.228					
Time^2^	0.097 [-0.071, 0.266]	0.263					
Time^3^	-0.149 [-0.318, 0.020]	0.090					

Model 1 is the random intercepts model for comparison purposes; model 2 is the random intercept + fixed linear, quadratic and cubic effect of time.

^a^ Family Burden Interview Schedule.

^b^ Confidence Interval.

^c^ p-value.

^d^ Coefficient of Determination.

^e^ Akaike Information Criterion.

^f^ Bayesian Information Criterion.

^g^ Log Likelihood.

^h^ Intraclass Correlation Coefficient.

**Figure 2:**
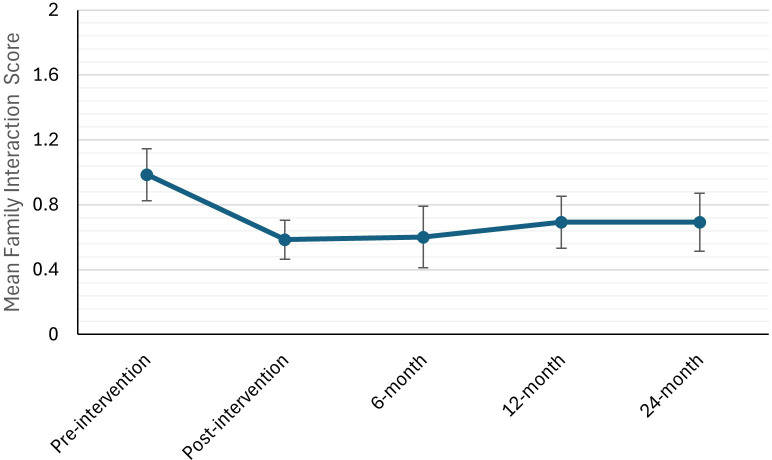
Mean family interaction burden aubscale scores across five time points (pre-intervention, post-intervention, 6-, 12-, and 24-month follow-up). Error bars represent standard error of the mean.

The linear mixed model predicting family interaction burden subscale scores also failed to demonstrate a significant main effect of time, *F* (4, 48) = 1.71, *p* = 0.162, *R²* = 0.0584 (**Table 3**; **Figure 2**). Exploratory *post-hoc* comparisons showed a statistically significant decrease in family interaction burden from pre- to post-intervention (*p* = 0.026), which persisted to the 6-month follow-up (*p* = 0.032). The decreases from pre-intervention to 12-month (*p* = 0.10) and to 24-month follow-ups (*p* = 0.10) were not significant. The quadratic effect of time in the model approached, but did not reach significance (*β* = 0.234, *p* = 0.063).

**Table 3:** Fixed effects of family interaction burden subscale across 24-month follow up.

Variable	Estimate [CI]^a^	p^b^	R^2c^	AIC^d^	BIC^e^	LogLikel.^f^	ICC^g^
Model 1			0.00	106.518	115.508	-51.493	0.405
Intercept	0.711 [0.478, 0.943]	<0.001					
Model 2			0.0584	107.5731	133.2232	-52.0012	0.421
Intercept	0.711 [0.478, 0.943]	<0.001					
Time	-0.151 [-0.392, 0.09]	0.226					
Time^2^	0.234 [-0.007, 0.476]	0.063					
Time^3^	-0.161 [-0.402, 0.081]	0.198					

Model 1 is the random intercepts model for comparison purposes; model 2 is the random intercept + fixed linear, quadratic, and cubic effect of time.

^a^ Confidence Interval.

^b^ p-value.

^c^ Coefficient of Determination.

^d^ Akaike Information Criterion.

^e^ Bayesian Information Criterion.

^f^ Log Likelihood.

^g^ Intraclass Correlation Coefficient.

## 4 Discussion

This pilot study assessed the preliminary outcomes of a psychoeducational group intervention designed to enhance the skills and knowledge of family carers supporting young adults with psychosis. No significant main effect of time was observed for overall family burden, indicating that the changes across the study period were not statistically significant at the model level. Exploratory *post-hoc* analyses showed a significant reduction in overall family burden pre-intervention to the 6-month follow-up point. In the subscale of family interaction burden, a significant decrease in burden was shown between pre- to post-intervention and was maintained at 6 months.

Participants reported benefits through feedback questionnaires, including increased knowledge and understanding about psychosis, feeling better supported in their caregiver needs, and learning from the lived experience of facilitators and other group members. The analysis of participant feedback also identified areas for potential program refinement, including delivery and pacing modifications, pathways to resources and exploration of particular areas of knowledge.

Taken together, both qualitative and quantitative findings hypothesize a beneficial impact of the psychoeducation intervention on family relational dynamics, which was a primary target of the intervention. While this pilot study does not have statistical power to derive clear conclusions regarding the effectiveness of the psychoeducational curriculum, a downward trend in overall burden indicates that the intervention warrants further investigation with larger samples.

These results are consistent with the growing body of literature supporting the effectiveness of family-based psychoeducational interventions in the care of young adults with psychosis, with family involvement increasingly recognized as key components of early psychosis recovery (10, 33, 34). The REACH program, a structured family psychoeducational intervention, demonstrated significant improvements in caregiver stress, family functioning, and reductions in expressed emotion among participating families across four months of follow-up (35). Similarly, NAMI’s Family-to-Family program, a peer-led intervention, has shown benefits from pre-intervention to post-intervention, including increased caregiver empowerment, knowledge, and coping (36).

Our intervention builds on this evidence base by integrating education about psychosis and its impact on families with two modalities which aim to reduce family burden and foster better communication within families: the LEAP^©^ technique (Listen, Empathize, Agree, Partner) and brief interventions derived from Acceptance and Commitment Therapy for Psychosis (ACTp) for carers (20–23). These approaches focus on improving relational dynamics, in particular coaching effective communication skills with persons who experience anosognosia, fostering empathy in carers toward both their young adult and themselves, and equipping carers with skills to more effectively respond to internal and external stressors.

Importantly, all modules were developed by teams of individuals with synergistic clinical expertise and family-lived experience, making the curriculum integrally co-produced. Similarly, the delivery of the curriculum uses a co-facilitation model that combines and coordinates the expertise of facilitators with lived experience as carers with the expertise of clinicians working in early psychosis intervention. Stories of lived experience are shared throughout the educational modules by facilitators, guest speakers, video media presentations, and the participants’ own contributions.

The results from the 24-month follow up pilot data of this novel approach to family psychoeducation, which synergizes clinical expertise with lived-experience expertise at all stages of curriculum development and implementation, generates momentum for exploring the curriculum effectiveness in a larger cohort.

## 5 Limitations

The limited sample size of the pilot study incurs a reduced statistical power of the analysis. As is typical of small pilot study cohorts, this study’s results must be considered preliminary because of the risk of imprecision in effect estimates and bias. A larger Phase II of the study will increase power to clarify long-term effects of the intervention across the different carer burden domains. It will also look at the effect of the psychoeducational intervention delivered to family carers on the health service utilization of the young adults affected by psychosis.

The study’s use of a pre-post design cannot, without randomization and a control group, determine the efficacy of the intervention. Observed changes may reflect natural recovery, regression to the mean, or other treatments and supports received during the study period rather than because of the intervention itself. A randomized controlled trial was not practical due to the challenges of withholding the intervention from families seeking it, and the inevitability of information sharing within the small community where the study was conducted (26).

Participant demographics showed disproportionate representation in the categories of sex (predominantly female), ethnicity (predominantly Caucasian), and participant education (predominantly had post-secondary education). Further exploration comparing general population demographics to those represented in the study cohort could generate an analysis as to whether the intervention is equally accessible to and or sought after by different segments of the population.

## 6 Implications for researchers and future research

This pilot study represents an initial, exploratory step in development, and early evaluation of a co-produced psychoeducational intervention for family carers supporting young adults with psychosis. The findings should be interpreted as exploratory and hypothesis-generating. Future research should employ more rigorous designs, including randomized controlled trials with appropriate comparison groups, larger sample sizes, and pre-specified primary and secondary outcomes. In addition, future studies should examine the sustainability of effects on burden over time and a potential mechanism of action.

Research may also examine how the program can be made more accessible within a healthcare system and within more diverse settings, the extent to which it can be scaled up and sustained over time, and how intervention fidelity can be maintained. Finally, future research may explore how this psychoeducational intervention can be integrated with complementary supports to better address the complex challenges that families face in supporting young adults with psychosis.

## Data Availability

The raw data supporting the conclusions of this article will be made available by the authors, without undue reservation.
