# Supplementary material for: Family psychoeducation to support patients with psychotic illness: two-year outcomes from a pre–post longitudinal pilot study
Source: Front Psychiatry. 2026 May 14;17:1724396. doi: 10.3389/fpsyt.2026.1724396 (PMC13218078; doi:10.3389/fpsyt.2026.1724396)
Supplement: Supplementary file 1 [file Table1.docx]

**Table 1. Demographic Characteristics of Pilot Sample**

| **Characteristic** |  | ***n*** | **% of sample** |
| --- | --- | --- | --- |
| Sex of Participant | Female | 10 | 77% |
|  | Male | 3 | 23% |
| Ethnicity of Participant | Black | 3 | 23% |
|  | Indigenous | 1 | 8% |
|  | South Asian | 1 | 8% |
|  | White | 8 | 62% |
| Sex of Young Adult with Psychosis | Female | 2 | 15% |
|  | Male | 11 | 85% |
| Relationship of Young Adult to Participant | Child | 10 | 77% |
|  | Grandchild | 2 | 15% |
|  | Niece/Nephew | 0 | 0% |
|  | Sibling | 1 | 8% |
| Participant Education | High School | 1 | 8% |
|  | College, CEGEP or other non-university certificate | 7 | 54% |
|  | University Diploma/certificate | 4 | 31% |
|  | Doctoral Degree | 1 | 8% |
| Employment Status | Casual | 1 | 8% |
|  | Full-time | 10 | 77% |
|  | Self-employed | 1 | 8% |
|  | Retired | 1 | 8% |
| Marital Status | Married | 9 | 69% |
|  | Divorced | 2 | 15% |
|  | Widowed | 1 | 8% |
|  | Other | 1 | 8% |
| Participant Difficulty Meeting Basic Needs | Yes | 1 | 8% |
|  | No | 12 | 92% |
| Presence of Other Supports | Yes | 11 | 85% |
|  | No | 2 | 15% |
| Young Adults’ Living Situation | Within Family’s Home | 9 | 69% |
|  | Own Home | 3 | 23% |
|  | Other | 1 | 8% |
